# The People’s Trial: supporting the public’s understanding of randomised trials

**DOI:** 10.1186/s13063-021-05984-1

**Published:** 2022-03-09

**Authors:** Elaine Finucane, Ann O’Brien, Shaun Treweek, John Newell, Kishor Das, Sarah Chapman, Paul Wicks, Sandra Galvin, Patricia Healy, Linda Biesty, Katie Gillies, Anna Noel-Storr, Heidi Gardner, Mary Frances O’Reilly, Declan Devane

**Affiliations:** 1 School of Nursing and Midwifery, National University of Ireland Galway, Galway, Ireland; 2 Health Research Board-Trials Methodology Research Network, School of Nursing and Midwifery, National University of Ireland Galway, Galway, Ireland; 3 Evidence Synthesis Ireland, School of Nursing and Midwifery, National University of Ireland Galway, Galway, Ireland; 4 J.E. Cairnes School of Business & Economics, National University of Ireland Galway, Galway, Ireland; 5 Health Services Research Unit, Health Sciences Building, University of Aberdeen, Foresterhill, Aberdeen, AB25 2ZD UK; 6 School of Mathematics, Statistics and Applied Mathematics, National University of Ireland Galway, Galway, Ireland; 7 Cochrane UK, hosted by Oxford University Hospitals NHS Foundation Trust and funded by the National Institute for Health Research, Oxford, UK; 8 Wicks Digital Health, Lichfield, England, UK; 9 Radcliffe Department of Medicine, University of Oxford, Oxford, UK; 10 Formerly - Nursing and Midwifery Planning and Development Unit, West Mid-West, Merlin Park University Hospital, Galway, Ireland; 11 Cochrane Ireland, National University of Ireland Galway, Galway, Ireland

**Keywords:** Randomised trial, Public engagement, Online, Methodology

## Abstract

**Background:**

Randomised trials are considered the gold standard in providing robust evidence on the effectiveness of interventions. However, there are relatively few initiatives to help increase public understanding of what randomised trials are and why they are important. This limits the overall acceptance of and public participation in clinical trials. *The People’s Trial* aims to help the public learn about randomised trials, to understand why they matter, and to be better equipped to think critically about health claims by actively involving them in all aspects of trial design. This was done by involving the public in the design, conduct, and dissemination of a randomised trial.

**Methods:**

Using a reflexive approach, we describe the processes of development, conduct, and dissemination of *The People’s Trial*.

**Results:**

Over 3000 members of the public, from 72 countries, participated in *The People’s Trial*. Through a series of online surveys, the public designed a trial called The Reading Trial. They chose the question the trial would try to answer and decided the components of the trial question. In December 2019, 991 participants were recruited to a trial to answer the question identified and prioritised by the public, i.e. ‘*Does reading a book in bed make a difference to sleep in comparison with not reading a book in bed?*’ We report the processes of *The People’s Trial* in seven phases, paralleling the steps of a randomised trial, i.e. question identification and prioritisation, recruitment, randomisation, trial conduct, data analysis, and sharing of findings. We describe the decisions we made, the processes we used, the challenges we encountered, and the lessons we learned.

**Conclusion:**

*The People’s Trial* involved the public successfully in the design, conduct, and dissemination of a randomised trial demonstrating the potential for such initiatives to help the public learn about randomised trials, to understand why they matter, and to be better equipped to think critically about health claims.

**Trial registration:**

ClinicalTrials.govNCT04185818. Registered on 4 December 2019

**Supplementary Information:**

The online version contains supplementary material available at 10.1186/s13063-021-05984-1.

## Background

Randomised trials are an important research design in evaluating the effects of health interventions. They have the potential to provide reliable evidence to inform health decisions. While these are exciting and challenging times for clinical trials, rising costs and regulations are making trials more expensive and complicated. For example, a study undertaken by the US Bureau of Labor Statistics found the mean cost per patient in clinical trials quadrupled between 1989 and 2011—increasing from $3773 to $16,567 [1].

Substantial amounts of public and charitable funding are allocated to clinical research every year [2]. There are, however, serious concerns that much of this is wasted [3]. The reasons for this waste include failure to publish completed research, inadequate reporting of research, and the development of new studies without placing them in the context of previous research addressing the same question [3, 4]. Also, inadequate recruitment and retention of participants to trials lead to waste due to trials not being able to provide sufficient high-quality evidence to answer the question for which it was designed [ 5, 6]. The inability to recruit enough participants to answer a trial question is one of the main reasons trials are discontinued or request extensions, with just over 50% of trials meeting their pre-specified recruitment targets [6, 7].

It is important to understand why members of the public consider participating, or not participating, in a trial [6, 8]. A 2017 survey of over 12,000 members of the public, from 68 countries, including 2194 clinical trial participants, found that 84% of respondents perceived clinical research to be important, while 82% reported that they felt well informed about clinical research [9, 10]. However, more detailed results demonstrate that public knowledge and understanding of clinical research may be superficial, with 51% of respondents, across five continents, reporting that they do not know where research is conducted and 34% of respondents reporting that they do not know what percentage of medicines must be tested in clinical research studies before being sold to the public [9, 10]. No differences in responses were noted when participants’ country of domicile was considered. Troublingly, the proportion of people ‘very willing’ to participate in a clinical trial was significantly lower (31%) than a similar survey conducted by the same group 4 years previously (50%) [9]. Respondents who felt informed about clinical research were more willing to participate in clinical trials. When asked how the public should be educated about the clinical research process, 35% of respondents proposed learning about clinical research through educational information on the internet [9].

Fear and distrust of research have been described as barriers to public involvement in research [11]. This was found to be more common within underserved groups, such as ethnic minorities, with further systematic reviews highlighting mistrust in research as a barrier to recruitment of vulnerable populations [12, 13]. The findings of these reviews suggest that fear and mistrust of research are linked to a lack of knowledge and understanding of clinical research or the research process. While these reviews found that knowledge had a positive impact on recruitment to clinical research, they also highlighted that confusion or a lack of understanding around specific trial processes, such as randomisation, acted as barriers to recruitment, particularly in obtaining informed consent [8, 11, 12, 14].

This evidence suggests a lack of understanding of what randomised trials are and why they are essential. This may negatively affect public support for and participation in clinical trials [15, 16]. A poor understanding of evidence may lead to public health risks such as the under or overuse of medicines, uninformed health choices, and unnecessary human suffering [17]. In recent times, there has been a sustained effort by stakeholders, including funders, policymakers, and patient and public involvement and engagement partners to involve members of the public in all aspects of research to support engagement with and participation in clinical trials [18–20]. Yet, to date, relatively few initiatives (i.e. The Informed Health Choices [21], Just Ask 2020 [22]) have been developed to support the general public’s understanding of randomised trials. *The People’s Trial* is one such initiative. This paper reports the process of *The People’s Trial*, which actively involved the public in the design, conduct, and dissemination of a randomised trial to support understanding of randomised trials.

## Methods/design

### Aim

The *People’s Trial* aimed to help the public learn about randomised trials, to understand why they matter, and to be better equipped to think critically about health claims.

### Study design and setting

*The People’s Trial* was an online trial, designed by the people for the people. Using a custom-built, online platform, it sought to involve the public in all steps of a randomised trial.

### Theoretical perspective

*The People’s Trial* embraced the concept of ‘learning by doing’. It sought to enhance understanding of randomised trials by facilitating the involvement of the public in the trial research process. Malcolm Knowles’ theory of andragogy informed the design of *The People’s Trial* [23]. Andragogy focuses specifically on the ways adults learn. Knowles believed that adult learning should involve collaborative interactions, including the use of available resources. Knowles identified five assumptions that encourage successful adult learning (see Table 1). These assumptions were incorporated into the design of *The People’s Trial*.

**Table 1:** Malcolm Knowles’ theory of andragogy five assumptions

Assumptions	
Self-concept	Adult learners have an established sense of self-value and autonomy and benefit from active involvement in their learning.
Experience	Adult learners bring a lifetime of experience. To stimulate and maintain interest, participant’s life experiences should be, engaged with, and connected to during the learning process.
Readiness to learn	Readiness to learn stems from adult learners recognising and appreciating the intrinsic value of their newly acquired knowledge.
Learning orientation	Adults learn best through practical application or “learning by doing”.
Motivation to learn	Adult learners are generally motivated to learn by internal factors (i.e. self-esteem and self-value) rather than external factors (for example, a pay increase).

### The team and working process

We conducted *The People’s Trial* on a custom-designed, online platform (www.ThePeoplesTrial.ie). We divided the trial into seven phases, paralleling the process of a randomised trial, i.e. (i) question identification, (ii) question prioritisation and selection, (iii) determining how we would answer the trial question, (iv) recruitment and randomisation, (v) trial conduct and data analysis, (vi) developing a dissemination strategy, and (vii) dissemination of trial findings [24, 25]. Doing so would, we felt, open the trial methodology process to the public. We used plain language text in all communications to maximise the accessibility of the trial to the public. All text was reviewed by our steering group, which included research communicators and members of the public. We produced a series of animated whiteboard videos for each phase. These animations explained each step of the trial process as it progressed and were narrated by researchers, clinicians, and members of the public. All were designed and produced to be accessible and engaging.

During each phase of *The People’s Trial*, we collected and reported website analytics, media, and social media metrics. Our hosting platform captured survey participation metrics.

We established a steering group of trialists, methodologists, statisticians, clinicians, research communicators, and members of the public to oversee *The People’s Trial*. Collectively, this group supported the methodological decisions and processes of the trial with a priority focus on ensuring public involvement in the trial processes from question identification and prioritisation to dissemination of trial findings.

### Participants

From April 2019, utilising a broad social media campaign, the public was invited to take part in the different phases of *The People’s Trial*. Members of the public were invited to participate in each phase of the trial independently and could take part in some or all of the phases, as they wished (Fig. 2). Participants in *The People’s Trial* were 18 years of age or over. As we were unable to offer a translation service, participants also needed to be able to communicate in English and give written informed consent.

### Procedures

#### Pre-launch

We used social media campaigns to create awareness of *The People’s Trial*, highlighting the motivation behind the trial. We promoted *The People’s Trial* through engaging custom-designed animations, which guided the public through the framework and objectives of the trial, highlighting the opportunity for shared learning. We also targeted national media (radio, newspaper, and TV networks) with press releases promoting *The People’s Trial.* During this time, *The People’s Trial* website introduced the public and participants to members of the steering group and the collective expertise they brought to the project.

In preparation for the trial launch, we provided accessible, exemplar questions that trials could answer and would be familiar with a broad public audience, such as, *Does eating cheese cause nightmares compared to not eating cheese*? We explained that only fun, accessible questions would be accepted to ensure the trial was low-risk and accessible to all members of the public.

#### Baseline data

*The People’s Trial* was conducted in phases, mimicking the steps of a randomised trial, and took place over a period of 10 months commencing in April 2019. The public was invited to participate in each phase of *The People’s Trial *and could choose to take part in some, or all of the phases, as they wished.

We invited participants to read an information leaflet about the study and sought their consent to participate through a custom-designed online form. This online information leaflet advised that participation in *The People’s Trial* was voluntary, and participants were free to withdraw from the trial at any time. We asked each participant to provide baseline data on whether they worked in healthcare or health research, what their understanding of randomised trials was before taking part in *The People’s Trial*, and their age and gender. All information submitted by the public was confidential and anonymised.

#### Phase one: All good trials start with a good question

**Fig. 1:**
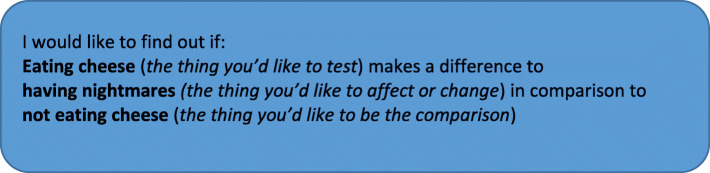
Exemplar question

In phase one, we invited the public to submit a question they would like *The People’s Trial* to tackle using a randomised trial design. We asked the public to submit their question on a QuestionPro® form embedded on *The People’s Trial* website. We structured the form, and gave examples, using the framework of intervention, comparator, and outcome (Fig. 1). All software, surveys and questionnaires used throughout *The People’s Trial* were piloted by volunteer testers, including members of the public, researchers, and steering group members prior to public release. In addition, an animated video offered further insights into what makes a good research question and why this process is important.

#### Phase two: A good question is one that people want to know the answer to

During phase two, we developed two surveys to investigate how little, or how much, the public liked each question submitted in phase one.

Two steering group members reviewed all the questions submitted in phase one. A third member reviewed the questions where an initial consensus on inclusion was not reached. If necessary, these questions were discussed further by the steering group. We excluded the questions where the outcome was a health outcome requiring medical assessment and questions that targeted participants with a medical condition. We provided specific feedback on the reasons why a question was not included and posted this information on *The People’s Trial* website. During this phase, we also introduced the concept of research waste and the potential cost and ethical implications associated with it.

##### Survey 1

Using a 3-point sliding scale ((i) *no thanks, I’m not interested in us answering this question*; (ii) *I’m not sure*; and (iii) *Yes please, I’m really interested in us answering this question*), participants indicated how interested they were in answering each question. We then ranked the questions based on these preferences. Using this method, the public selected the top ten most popular questions.

##### Survey 2

In survey 2, using a click and drop method, the members of the public ranked the top ten questions in order of preference (question ranked number 1 = most favourite, and question number 10 = least favourite)

Using this online iterative process, the question chosen by the public for *The People’s Trial* to tackle was:

*Does reading a book in bed make a difference to sleep in comparison with not reading a book in bed*?

#### Phase three: We have our question... Now it’s time to think about how we’ll answer this question

In phase three, we asked the public to determine the characteristics of the intervention (reading a book in bed), the comparator (not reading a book), and the outcome (sleep). For example, we asked the public to decide if trial participants should ‘Have the use of electronic entertainment or communication devices (e.g. mobile phones /tablets) in bed?’, ‘Go to bed and wake up at the same time as they normally would?’, and ‘Sleep in their own bed, in their own home, for the study duration’. We also asked the public to tell us how they felt we should measure the outcome of ‘sleep’. Again, an animated video described the importance of this step in trial design.

Through this process, the public defined the intervention and comparator and decided that the primary outcome should be an evaluation of overall sleep quality, with daytime sleepiness and sleep disturbance assessed as secondary outcomes.

#### Phase four: Being a bit random—deciding who gets what in the trial?

In phase four, we invited the public to take part in *The People’s Trial* randomised trial, which we called ‘The Reading Trial’. On December 4, 2019, we began to recruit members of the public who were, 18 years of age or older, to take part in the trial. Recruitment remained open until January 1, 2020, with public engagement maintained using a broad social media campaign. After they provided consent, participants clicked a button to self-randomise to either the intervention or the control group using a custom-built, randomisation tool, developed by Metaxis Software design® embedded in *The People’s Trial* website. An engaging, animated video explained ‘randomisation’, and why it is important in clinical trials.

#### Phase five: Does reading a book in bed make a difference to sleep?

During phase five, we conducted ‘The Reading Trial’, an online, parallel-group, randomised trial, designed by the people, for the people. Participants self-randomised and were allocated in a 1:1 ratio, to the intervention group (reading a book in bed) or the control group (not reading a book in bed). We registered the ‘Reading Trial’ before recruitment of the first participant (registration number: NCT04185818). This trial was conducted solely online; we utilised social media to support intervention fidelity through regular online engagement with participants. The primary outcome of The Reading Trial was overall sleep quality, measured using a self-reported scale called the ‘single item sleep quality scale (SQS)’ [26]. Secondary outcomes were sleep disturbance and daytime sleepiness measured using self-reported scales [27, 28]. For all outcomes, the time frame we analysed was the 7 days the participant took part in The Reading Trial. Further details are included in the trial report available here.

#### Phase six: Reporting what we found

Funders and regulatory bodies require that trial results are made available to all stakeholders in a timely and accessible manner. An effective dissemination strategy leads to an increased awareness of the research being undertaken, promotes discussion, and highlights potential health benefits to stakeholders. Also, accessible and usable reporting of trial results increases the value and minimises avoidable research waste. However, most research dissemination is typically limited to academic and professional journals, which the public may not have access to or even be aware of. To ensure our trial results were accessible to the general public, our target audience, we used an online survey to ask participants of ‘The Reading Trial’ to rank in order of importance how and where they would like to see the results of the trial publicised.

#### Phase seven: So what have we learnt?

During this phase, we reported the findings of ‘The Reading Trial’. The dissemination strategy used was directly informed by the results of the phase six, online survey. The trial report is available here.

## Results

**Fig. 2:**
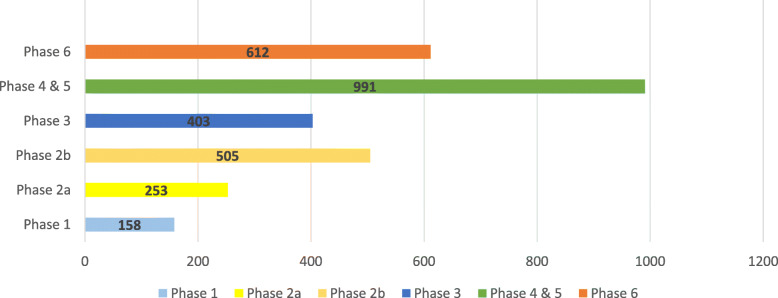
*The People’s Trial* participant numbers by phase

*The People’s Trial* was conducted, between April 2019 and November 2020. Over 3000 members of the public, from 72 countries, took part in *The People’s Trial* (Fig. 2). Participants were invited to take part in each phase independently, meaning that individuals could participate in more than one phase, indeed this was encouraged.

### Phase one

During phase one, the public submitted 155 potential questions for *The People’s Trial* to answer. Almost half (*n* = 67, 43%) of participants in this phase described themselves as having ‘none’ or ‘some’ understanding of randomised trials before taking part in *The People's Trial*.

### Phase two

In phase two, we reviewed the 155 questions submitted by the public during phase one. We excluded 99 questions where the outcome was a health outcome requiring medical assessment and questions that targeted participants with a medical condition. We also merged similar questions where possible. This process produced 41 questions. We prioritised the questions in two surveys.

#### Survey 1

During survey 1, 253 participants selected their top ten favourite questions from these 41 questions (see supplementary file 1). This survey found that the question *Does reading a book in bed make a difference to sleep in comparison with not reading a book in bed?* was rated highly by participants, with 59% (*n* = 117) reporting that they were ‘really interested’ in answering the question.

#### Survey 2

During survey 2, 505 members of the public ranked the top ten questions in order of preference (Table 2). The question ranked number 1 by the largest percentage of participants (19%, *n* = 97) was *Does reading a book in bed make a difference to sleep in comparison with not reading a book in bed? This was the question The People's Trial tackled.*

**Table 2:** Top ten questions chosen by the public

Rank	Percentage of votes received	Question
1	19%	Does reading a book in bed make a difference to sleep in comparison with not reading a book in bed?
2	14%	Does using a mobile phone before sleeping make a difference to sleep quality in comparison with not using a mobile phone before sleeping?
3	12%	Does doing daily crosswords or puzzles make a difference to your memory in comparison with not doing daily crosswords or puzzles?
4	10%	Does exercising right after waking up make a difference to productivity at work in comparison with not exercising right after waking up?
5	10%	Does eating breakfast make a difference to concentration in the mornings in comparison with not eating breakfast?
6	9%	Does not viewing social media make a difference to short-term mood in comparison with viewing social media?
7	9%	Does going for a walk outside at lunchtime make a difference to concentration in the afternoon in comparison with not going for a walk at lunchtime?
8	6%	Does light exercise in the evening make a difference to sleep quality in comparison with no exercise in the evening?
9	6%	Does outdoor exercise make a difference to short-term mood in comparison with indoor exercise?
10	5%	Does spending time outdoors make a difference to short-term mood in comparison with not spending time outdoors?

### Phase three

During phase three, we asked the public to consider how we could answer this question. Following a broad media campaign including social media, traditional print media, and national radio stations, 403 members of the public responded to our online survey to determine the characteristics of the intervention (reading a book in bed), the comparator (not reading a book in bed), and the outcome (sleep).

The public decided that participants randomised to the intervention group (reading a book in bed) should:

1. Read a book immediately before trying to go to sleep
2. Read for 15–30 minutes
3. Go to bed and wake up at the same time as they usually would
4. Not eat food or drink caffeinated drinks within 1 hour of going to bed
5. Sleep in their bed, in their own home

This should be done for the study duration (7 nights).

Similarly, participants in the control group (not reading a book in bed) should:

1. Go to bed, and wake up at the same time as they normally would
2. Not eat food or drink caffeinated drinks within 1 hour of bed
3. Sleep in their bed, in their own home, for the study duration (7 nights)

However, as the control group, these participants should **not** read a book immediately before trying to go to sleep.

The public also decided that participants in both groups could use electronic entertainment or communication devices (e.g. mobile phones /tablets) in bed for participants in both the intervention and control groups.

The only difference between the intervention group and the control group was reading a book in bed for the study duration (7 nights).

### Phase four

Between 4th December 2019 and 31st December 2019, 991 people agreed to take part in The Reading Trial. Of these, 496 (50%) were allocated by chance to the intervention group (reading before sleeping) and 495 (50%) to the control group (not reading before sleeping).

Although 564 participants were required to achieve a priori sample size, the primary aim of *The People’s Trial* was to help the public learn about randomised trials, so we continued enrolment after this sample size was achieved.

The reading trial had an attrition rate of 21.9% (*n* = 217). Of those that did not finish the study, 127/496 (25.6%) were in the ‘intervention’ group (reading a book before sleeping) and 90/495 (18.2%) in the control group (i.e. not reading a book before sleeping).

**Fig. 3:**
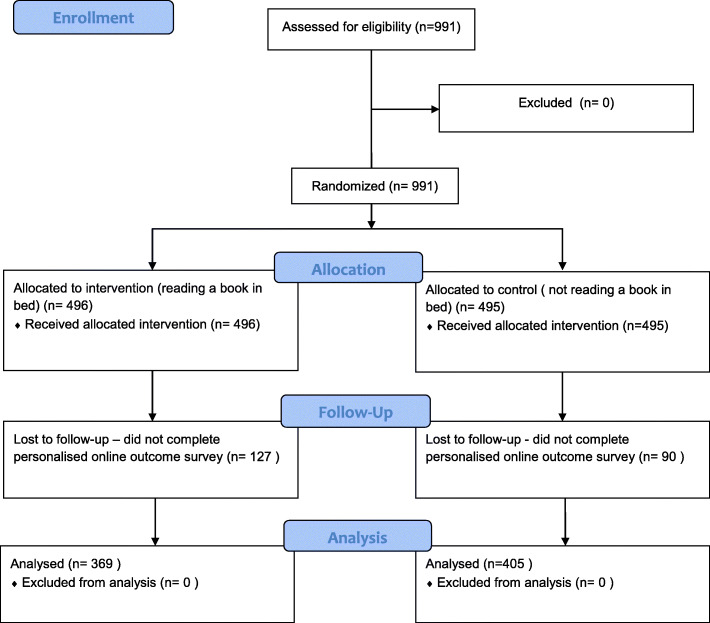
The Reading Trial consort flow diagram

However, 774 people (369 (47.7%) people in the intervention group and 405 (52.3%) in the control group) from 43 countries reported outcomes (Fig. 3).

The two groups were, on average, similar in baseline characteristics. Also, participants in both groups reported similar sleep quality at the beginning of the trial (Table 3).

**Table 3:** Summary statistics for participant characteristics at the start of ‘The Reading Trial’

People who took part in The Reading Trial	Reading group (*n* = 369), *n* (%)	Not reading group (*n* = 405), *n* (%)
Age		
18–24 years	21 (6%)	28 (7%)
25–44 years	193 (52%)	209 (51%)
45–64 years	123 (33%)	145 (36%)
65 years and over	32 (9%)	23 (6%)
Gender		
Female	289 (78%)	325 (80%)
Male	75 (20%)	78 (19%)
Prefer not to say/ self-describe	5 (1%)	2 (0.5%)
Understanding of randomised trials		
Good understanding	251 (68%)	278 (69%)
Some understanding	101 (27%)	105 (26%)
No understanding	17 (5%)	22 (5%)
Healthcare background		
Healthcare	238 (65%)	269 (66%)
Not healthcare	131 (34%)	136 (34%)
Sleep quality at the start of the trial		
Terrible	7 (2%)	6 (1%)
Poor	51 (14%)	51 (13%)
Fair	175 (47%)	181 (45%)
Good	115 (31%)	152 (37%)
Excellent	21 (6%)	15 (4%)

Data are numbers of people (%)

### Phase five

The reading trial found that reading a book in bed before going to sleep improved sleep compared to not reading a book in bed before going to sleep. In the intervention group, 156 (42%) people reported an improvement in their sleep quality compared to 112(28%) people in the control group, a difference of 14% favouring the intervention group. Considering the uncertainty in this estimate, we calculated that the difference in the population is likely to be between 8 and 22%, favouring those on the intervention. The full results of The Reading Trial are reported separately as a plain language trial report.

### Phase six

**Fig. 4:**
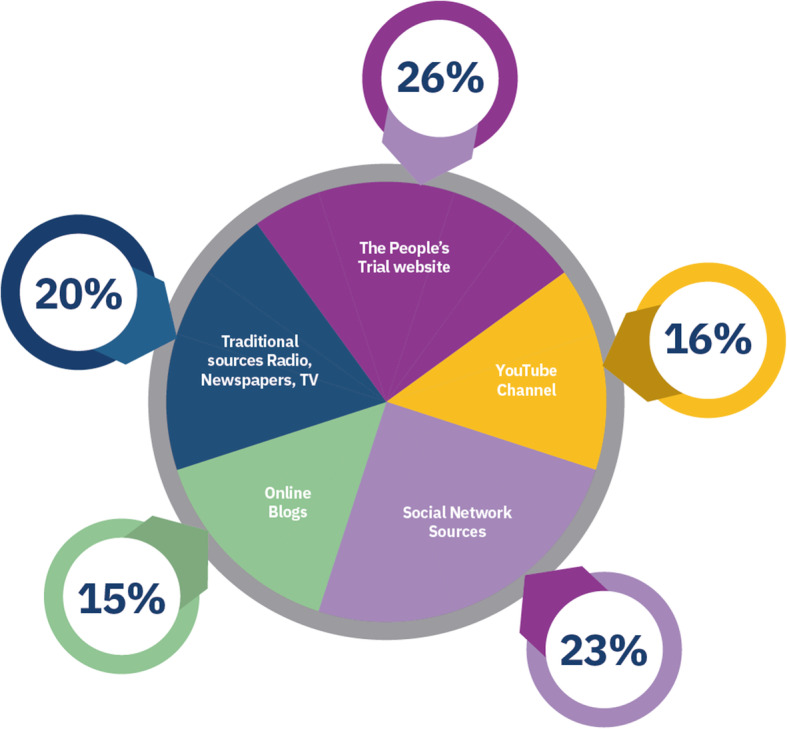
Where the public want the results disseminated

**Fig. 5:**
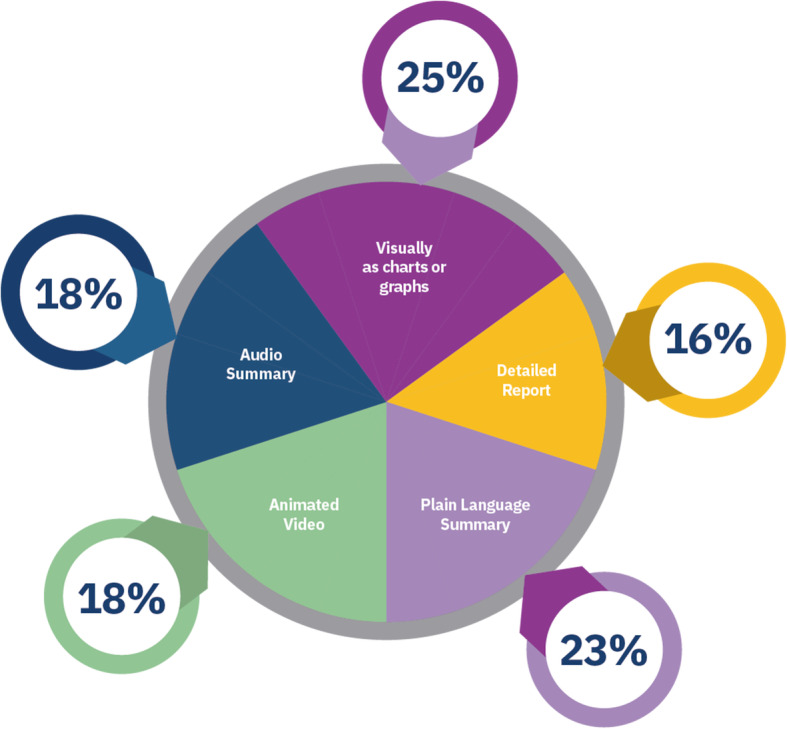
How the public want the results disseminated

During phase six, 612 participants, from 47 countries, told us where and how they would like the results of The Reading Trial publicised (Figs. 4 and 5). Most people chose to have the trial results displayed visually or published as a plain-language summary (Fig. 5). The public indicated they would like to see the results displayed on *The People’s Trial* website and publicised through social media campaigns (Fig. 4). The findings of this online survey informed *The People’s Trial* dissemination strategy, which includes a plain-language report, the publication of all trial results on *The People’s Trial* website visually, through graphs, an animated short video, and an audio blog. *The People’s Trial* website, including the results of The Reading Trial, is maintained as a live site and is freely accessible to all. In addition, access to individual predictive results using a custom-designed nomogram, embedded on *The People’s Trial* website is in development and will be available to all on the website.

*The People’s Trial* website (www.thepeoplestrial.ie) has recorded 9,552 unique users, with 15,258 sessions and 25,382 page views to date.

Visitors to the site were predominantly from Ireland (35%) and the UK (35%) (see Table 4). Overall, 46% of site users were female, and 54% were male.

**Table 4:** *The People’s Trial* website demographics

Country	Number of unique visitors to www.Thepeoplestrial.ie
Ireland	3205 (35%)
UK	3198 (35%)
USA	376 (4%)
France	308 (3%)
Australia	246 (3%)
Germany	232 (3%)
Canada	231 (3%)
Russia	101 (1%)
India	79 (0.86%)
Spain	78 (0.85%)

**Fig. 6:**
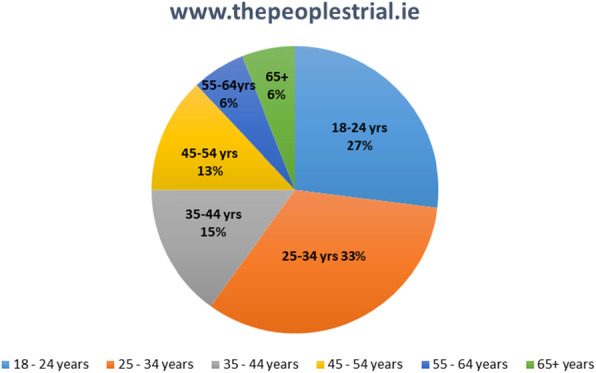
Age profile of website users

While *The People’s Trial* website attracted users from all age groups, 61% were between 18 and 34 years, with just 6% of users age 65 or older (Fig. 6).

Social media was the principal method of advertising for *The People’s Trial*, with 39% of new users accessing *The People’s Trial* website directly from social media platforms. During the recruitment phase of The Reading Trial (December 2019), *The People’s Trial* Twitter account achieved 197k Twitter impressions, with 3029 profile visits and 275 mentions. Traditional media sources were also utilised to promote the trial, incorporating press releases, blogs, and interviews with members of the steering group on national radio stations.

### Continued accessibility

*The People’s Trial* website (www.ThePeoplesTrial.ie) is maintained as a live site with unrestricted, public access to review all steps of the trial, including the results of The Reading Trial. All educational tools, such as the animated explanatory videos, are maintained on this site and *The People’s Trial* YouTube channel, and are free to use.

## Discussion

While clinical trials are not unusual, and there have been initiatives which aim to educate members of the public about randomised trials, such as the ‘Understanding Clinical Trials’ programme funded by CISCRP, or the ‘Wellcome Monitor’, funded by the Wellcome Trust, we believe *The People’s Trial* is unique in its active involvement of the public in creating the steps of the trial process from identifying and prioritising the trial question to trial conduct and dissemination [29, 30]. *The People’s Trial* offered members of the public the opportunity to take part in and learn about randomised trials in an accessible, online environment. The trial supported a shared learning experience for participants and researchers, where members of the public were supported to learn about randomised trials through active participation in all trial processes. In addition, researchers gained further understanding on how public participation could inform and improve trial processes. This project demonstrates a public willingness to access and engage with learning and knowledge on trial methodology. *The People’s Trial* highlights the role social media can play in promoting clinical trials and their processes within the broader public arena, supporting public engagement and participation in future clinical trials. An evaluation of the experiences of participants in *The People’s Trial *has been conducted and is under analysis. The findings of this evaluation will support future public participation in the design and conduct of randomised trials and will be available on *The People’s Trial* website.

While an online trial supports accessibility and inclusion, it was not without its challenges, primarily because of the nature of *The People’s Trial*. *The People’s Trial* began as a concept, which required the active participation of members of the public to develop. When designing *The People’s Trial* website, the steering group did not know the trial question, and therefore, the intervention, comparator, outcome, sample size, etc. were all unknown. The team, including our web development team, had to respond organically, and promptly, to the trial needs as it progressed. To minimise the risk of project slippage, we engaged experienced web designers and volunteer testers to ensure all aspects of the online trial were fully functional before releasing each phase to the public. To further promote inclusion, the website design was optimised specifically for members of the public participating on mobile phones.

Due to budget constraints, a significant limitation of the trial was the exclusion of individuals not competent in the English language. While we would like to see this limitation addressed in future trials, all members of the steering group worked to ensure the language used throughout *The People’s Trial* was accessible, appropriate, and relevant, albeit in English only.

A significant unforeseen challenge to *The People’s Trial* was the current COVID-19 pandemic. Although the trial conduct and data collection were completed before the onset of the pandemic, the publication of the results of ‘The Reading Trial’ and the invitation to participate in the evaluation survey was delayed. The delay was primarily due to the re-assignment of the research team to research projects focused on the coronavirus pandemic.

## Conclusion

To be effective, clinical trials need participants, but recruitment and retention continue to be challenging with almost half of all trials not meeting their target sample size [7]. The evidence suggests that knowledge of trials and why they are important has a positive impact on recruitment to clinical research [31]. While confusion and a lack of understanding of clinical trials have been shown to have the opposite effect [9, 12]. This paper describes the process of developing and conducting a novel, online initiative to potentially address these challenges.

In a time where the public is actively seeking information on trials and research methodology through online platforms, *The People’s Trial* offered the possibility of opening trial processes to a broader audience. *The People’s Trial* has demonstrated that social media platforms can be an invaluable tool in supporting future public engagement in research. The public’s views on trial design and the acceptability of trial processes have been underrepresented in research to date.

With over 3000 members of the public participating, from 72 different countries, *The People’s Trial* demonstrates the potential for public participation to inform and improve randomised trial processes, while also providing an opportunity for shared learning. *The People’s Trial* offers important insights for researchers on public involvement in designing trial processes. Using innovative, novel methods, it successfully engaged the broader public in planning, designing, conducting, and reporting a randomised trial.

## Supplementary Information

**Additional file 1.**

**Additional file 2.**

## Data Availability

All data and materials are available from the corresponding author on reasonable request.
