# Supplementary material for: The People’s Trial: supporting the public’s understanding of randomised trials
Source: Trials. 2022 Mar 9;23:205. doi: 10.1186/s13063-021-05984-1 (PMC8905031; doi:10.1186/s13063-021-05984-1)
Supplement: Supplementary file 1 — Additional file 1. [file 13063_2021_5984_MOESM1_ESM.docx]

**The People’s Trial** [**www.thepeoplestrial.ie**](http://www.thepeoplestrial.ie)

- **I would like to find out if** watching TV before sleeping **makes a difference to** sleep quality **in comparison to** not watching TV before sleeping?
- **I would like to find out if** using a mobile phone before sleeping **makes a difference to** sleep quality **in comparison to** not using mobile phone before sleeping?
- **I would like to find out if** drinking coffee **makes a difference to** sleep quality **in comparison to** not drinking coffee?
- **I would like to find out if** eating cheese before bedtime **makes a difference to** nightmares **in comparison to** not eating cheese before bedtime?
- **I would like to find out if** drinking warm milky drink before sleeping **makes a difference to** quality of sleep **in comparison to** not drinking a warm milky drink before sleeping?
- **I would like to find out if** having a bath or shower at night **makes a difference to** how quickly you fall asleep **in comparison to** just going to bed without taking a bath or shower
- **I would like to find out if** light exercise in the evening **makes a difference to** sleep quality **in comparison to** no exercise in the evening?
- **I would like to find out if** reading a book in bed **makes a difference to** sleep **in comparison to** not reading a book in bed?
- **I would like to find out if** wearing socks in bed **makes a difference to** how well you sleep **in comparison to** not wearing socks in bed
- **I would like to find out if** sleeping in complete darkness **makes a difference to** the length of time you sleep **in comparison to** not sleeping in complete darkness?
- **I would like to find out if** using scents (e.g. lavender, mint, etc.) at bedtime **makes a difference to** sleep **in comparison to** not using scents at bedtime?
- **I would like to find out if** not viewing social media **makes a difference to** short term mood **in comparison to** viewing social media?
- **I would like to find out if** limiting social media use to looking at videos of animals only **makes a difference to** short term mood **in comparison to** usual social media?
- **I would like to find out if** eating chocolate **makes a difference to** short term mood **in comparison to** not eating chocolate?
- **I would like to find out if** drinking coffee in a cafe/socially **makes a difference to** short term mood **in comparison to** drinking coffee as takeaway/on the run?
- **I would like to find out if** spending time outdoors **makes a difference to** short term mood **in comparison to** not spending time outdoors?
- **I would like to find out if** outdoor exercise makes **a difference to** short term mood **in comparison to** indoor exercise?
- **I would like to find out if** looking at pictures of landscapes **makes a difference to** short term mood **in comparison to** being outdoors?
- **I would like to find out if** listening to music **makes a difference to** relaxation **in comparison to** not listening to music?
- **I would like to find out if** taking cold showers in the morning **makes a difference to** short term mood **in comparison to** not taking cold showers in the morning?
- **I would like to find out if** getting out of bed on the wrong side (i.e. not your side of preference) **makes a difference to** short term mood **in comparison to** getting out of bed on your preferred side?
- **I would like to find out if** consuming caffeine **makes a difference to** concentration **in comparison to** not consuming caffeine?
- **I would like to find out if** eating breakfast **makes a difference to** concentration in the mornings **in comparison to** not eating breakfast?
- **I would like to find out if** doing puzzles **makes a difference to** concentration **in comparison to** not doing puzzles
- **I would like to find out if** going for a walk outside at lunchtime **makes a difference to** concentration in the afternoon **in comparison to** not going for a walk at lunchtime?
- **I would like to find out if** listening to rock music **makes a difference to** concentration **in comparison to** listening to classical music?
- **I would like to find out if** dancing to an upbeat song after lunch **makes a difference to** the ability to concentrate in the afternoon **in comparison to** going straight back to work after lunch without dancing?
- **I would like to find out if** doing daily crosswords or puzzles **makes a difference to** your memory **in comparison to** not doing daily crosswords or puzzles?
- **I would like to find out if** information in the way of song **makes a difference to** your memory in **comparison to** information given verbally (i.e., not through the medium of song)?
- **I would like to find out if** exercising right after waking up **makes a difference to** productivity at work **in comparison to** not exercising right after waking up?
- **I would like to find out if** playing high intensity games **makes a difference to** your reaction speeds **in comparison to** not playing high intensity games?
- **I would like to find out if** drinking coffee before one's breakfast **makes a difference to** the amount of food you eat in a day **in comparison to** having the first cup of coffee after eating one's breakfast?
- **I would like to find out if** drinking decaffeinated coffee **makes a difference to** makes you poop **in comparison to** drinking caffeinated coffee?
- **I would like to find out if** drinking coffee **makes a difference to** your energy **in comparison to** drinking tea?
- **I would like to find out if** biting a wooden spoon **makes a difference to** tears when chopping an onion **in comparison to** not biting anything / nothing in your mouth?
- **I would like to find out if** exercising with bare feet **makes a difference to** balance **in comparison to** exercising in shoes?
- **I would like to find out if** reading a newspaper headline **makes a difference to** people’s perception **in comparison to** reading the whole newspaper article?
- **I would like to find out if** performing a rain dance **makes a difference to** rain levels **in comparison to** not doing a rain dance?
- **I would like to find out if** bad news about climate breakdown **makes a difference to** people's desire to make changes to their lifestyles **in comparison to** information about what difference individual choices can make?
- **I would like to find out if** taking a deep breath before engaging in a difficult/passionate conversation **makes a difference to** nerves **in comparison to** just launching into that conversation?
- **I would like to find out if** sleeping naked **makes a difference to** how well you sleep **in comparison to** wearing pyjamas?
