# Supplementary material for: The People’s Trial: supporting the public’s understanding of randomised trials
Source: Trials. 2022 Mar 9;23:205. doi: 10.1186/s13063-021-05984-1 (PMC8905031; doi:10.1186/s13063-021-05984-1)
Supplement: Supplementary file 2 — Additional file 2. [file 13063_2021_5984_MOESM2_ESM.docx]

**The People’s Trial** [**www.thepeoplestrial.ie**](http://www.thepeoplestrial.ie)

- **I would like to find out if** going for a walk outside at lunchtime **makes a difference to** concentration in the afternoon **in comparison to** not going for a walk at lunchtime?
- **I would like to find out if** exercising right after waking up **makes a difference to** productivity at work **in comparison to** not exercising right after waking up?
- **I would like to find out if** reading a book in bed **makes a difference to** sleep **in comparison to** not reading a book in bed?
- **I would like to find out if** doing daily crosswords or puzzles **makes a difference to** your memory **in comparison to** not doing daily crosswords or puzzles?
- **I would like to find out if** light exercise in the evening **makes a difference to** sleep quality **in comparison to** no exercise in the evening?
- **I would like to find out if** using a mobile phone before sleeping **makes a difference to** sleep quality **in comparison to** not using mobile phone before sleeping?
- **I would like to find out if** eating breakfast **makes a difference to** concentration in the mornings **in comparison to** not eating breakfast?
- **I would like to find out if** spending time outdoors **makes a difference to** short term mood **in comparison to** not spending time outdoors?
- **I would like to find out if** not viewing social media **makes a difference to** short term mood **in comparison to** viewing social media?
- **I would like to find out if** outdoor exercise makes **a difference to** short term mood **in comparison to** indoor exercise?
